# The Mechanism of Zinc Oxide in Alleviating Diarrhea in Piglets after Weaning: A Review from the Perspective of Intestinal Barrier Function

**DOI:** 10.3390/ijms251810040

**Published:** 2024-09-18

**Authors:** Xiaopeng Tang, Kangning Xiong, Yan Zeng, Rejun Fang

**Affiliations:** 1 State Engineering Technology Institute for Karst Desertfication Control, School of Karst Science, Guizhou Normal University, Guiyang 550025, China; 201811001@gznu.edu.cn; 2 Key Laboratory for Information System of Mountainous Areas and Protection of Ecological Environment, Guizhou Normal University, Guiyang 550025, China; zengy91@126.com; 3 College of Animal Science, Hunan Agricultural University, Changsha 410128, China

**Keywords:** intestinal health, novel zinc oxide, piglets, post-weaning diarrhea, zinc oxide

## Abstract

Weaning is one of the most challenging phases for piglets, and it is also the time when piglets are the most susceptible to diarrhea, which may result in significant economic losses for pig production. One of the dietary strategies for reducing post-weaning diarrhea (PWD) in piglets is to provide them with a pharmacological dose of zinc oxide (ZnO). However, excessive or long-term usage of high-dose ZnO has significant impacts on pig health and the ecological environment. Therefore, caution should be exercised when considering the use of high-dose ZnO for the prevention or treatment of PWD in piglets. In this paper, the significant role of zinc in animal health, the potential mode of action of ZnO in alleviating diarrhea, and the impact of innovative, highly efficient ZnO alternatives on the regulation of piglet diarrhea were reviewed to offer insights into the application of novel ZnO in pig production.

## 1. Introduction

In modern intensive pig production, piglets are generally weaned between the ages of 21 and 28 days, which is beneficial to increase the number of piglets each year and decrease the vertical transmission of pathogens from the sow to the piglets, thus maximizing the sow’s reproductive performance [1,2]. Early weaning, however, will result in weaning stress due to the sudden separation from the sow, changes in diet from breast milk to solid meals, and changes in social structure [3,4,5]. Piglets within 3–4 weeks of age coincide with the period when maternal antibodies leave the piglets and there is insufficient antibody production in the piglets [6,7]. This period coincides with the time when the piglets are at their weakest immunocompetence, and this, combined with weaning stress, can easily lead to intestinal dysfunction, digestive disorders, and post-weaning diarrhea (PWD) in piglets [6,7,8,9,10].

Piglet diarrhea associated with weaning is becoming one of the most difficult diseases to control and is an urgent problem to be solved in pig production [10]. Weanling diarrhea is never caused by a single factor. The decrease in nutrient digestibility caused by intestinal injury is one of the root causes of diarrhea in weaned piglets. After weaning, piglets have to cope with the sudden absence of breast milk and switch to a plant-based solid diet that contains various antinutrients. At this time, the insufficient secretion of protease, lipase, and amylase in the gastrointestinal tract of piglets is attributed to the immature development of their gastrointestinal tract. Consequently, suboptimal digestion in piglets leads to the fermentation of undigested substances by microorganisms in the posterior intestine, which provides a good source of nutrients for some pathogenic bacteria to multiply, thereby disrupting the balance of the posterior intestinal flora [3]. Additionally, the ingestion of antigens presents in the diet, such as soybean antigens and other substances, triggers intestinal allergic reactions. Ultimately, these factors contribute to the occurrence of nutritional diarrhea in weaned piglets [9,11,12]. Intestinal damage caused by weaning stress can increase intestinal permeability, allow pathogenic microorganisms to more easily enter the body, and aggravate the diarrhea in piglets [3,13]. Enterotoxigenic *Escherichia coli* (*E. coli*) is a type of pathogen that can cause diarrhea in both newborn and weaned piglets [14,15]. Through the fimbriae on the surface of the bacteria, enterotoxigenic *E. coli* attaches to the intestinal mucosal epithelial cells and the distinct glycoprotein receptors of the surrounding mucosal layer and releases enough endotoxin to modify the intestinal water and electrolytes, resulting in diarrhea in piglets [16,17,18].

In production practice, a combination of nutritional strategies is often used to prevent or reduce diarrhea in weaned piglets, such as antibiotics [19,20,21], low protein diet technology [22,23,24], functional feed additives [25,26,27,28,29,30], and high-dose zinc (Zn) oxide (ZnO) [31,32]. Of these strategies, the use of high-dose ZnO after weaning has been shown to improve intestinal health, significantly reduce PWD, and enhance growth performance, including weight gain and the feed intake of piglets [31,32,33]. However, the long-term use of high doses of ZnO poses serious health and environmental risks, especially zinc pollution. Zn is a heavy metal with low bioavailability, requiring high doses to achieve its biological effects [34,35]. Due to the low absorption rate, most of the zinc is excreted with feces, causing heavy metal pollution when exposed to the environment [36,37]. With the continuous strengthening of national environmental awareness, it is not advisable to use high-dose ZnO to prevent or treat PWD in piglets. Therefore, it is important to summarize and understand how high-dose ZnO prevents diarrhea in piglets to identify effective substitutes with high availability and reduce Zn excretions. In this paper, the basic biological function of Zn, the mode of action of ZnO against diarrhea, and the effect of novel ZnO with high efficiency instead of traditional ZnO on controlling diarrhea in piglets were reviewed, which provided a theoretical basis for further Zn reduction in feed.

## 2. Important Role of Zinc in Piglet Health

Zn is one of the necessary trace elements in animals, which is involved in the synthesis and activation of proteins and related enzymes, bone development and keratinization, as well as the synthesis of transcription factors, and is related to a variety of physiological activities [38,39,40]. The Zn needed by animals is obtained through diet or drinking water. Zn is widely distributed in animals and has tissue differences, mainly distributed in muscle (approximately 60%), bone (approximately 30%), liver and skin (approximately 30%), and other tissues (about 2–3%) [41].

Extensive research has been conducted to investigate the significance of Zn in animal health (Table 1). Zn, as a vital trace element, plays a crucial role in the growth and development of animals, and its important role is reflected in many aspects. Firstly, Zn is involved in the composition of many enzymes in animals, or as an activator of enzymes, and is closely related to the activity and function of more than 300 enzymes, including six major enzyme classes (oxidoreductase, transferase, hydrolase, lyase, isomerase, and ligase) [42]. On the one hand, Zn acts as an enzyme component or regulatory factor to regulate cellular processes; on the other hand, it also participates in signal transduction as a signal molecule, regulating physiological processes and functions such as animal growth, redox, immunity, metabolism, and reproduction [43,44,45]. Secondly, Zn is also involved in gene regulation via Zn-finger proteins (ZFPs), which act as a transcriptional suppressor and activator of multiple genes, thereby regulating a variety of biological processes [41,46,47,48]. In pig production, weaning may lead to Zn deficiency in piglets, as confirmed by Davin et al. [49], who reported that the body Zn content of weaned piglets was lower than that of non-weaned piglets and the addition of ZnO can significantly increase the Zn content of weaned piglets. Zn deficiency can have serious negative effects on piglets, such as malnutrition, skin hyperkeratosis, growth retardation, and feed refusal, and ultimately affect the production performance and antistress ability of piglets [50,51]. Zinc deficiency in weaned piglets is more likely to lead to diarrhea, as demonstrated by Hassen et al. [34] who reported that the risk of diarrhea increased up to 60% for pigs that had a blood Zn concentration that decreased after weaning. However, the exact pathophysiological mechanism between diarrhea and zinc deficiency has not been clarified. The positive impact of pharmacological doses of ZnO on preventing or treating PWD in piglets has been fully demonstrated [31,33,52,53]. Therefore, understanding the mode of action of how ZnO controls diarrhea in piglets would provide important information for finding alternatives or reducing the high doses currently used. The following presents a summary of the potential mode of action of ZnO regulation in piglet diarrhea.

## 3. Possible Mechanism of ZnO to Prevent Diarrhea in Piglets

### 3.1. Antibacterial Effect of ZnO

Pathogenic bacterial infection is one of the main causes of diarrhea after weaning, and it can cause an increase in infectious disease and mortality in piglets [75]. Therefore, inhibiting the growth of harmful microorganisms by nutritional strategies can effectively control piglet diarrhea. Numerous studies have demonstrated that ZnO has a very strong antibacterial or bactericidal function [76,77,78]. For instance, Liu et al. [79] demonstrated that ZnO nanoparticles (ZnO-NPs) possess antibacterial properties against *E. coli* O157:H7, which suggested that ZnO-NPs have the potential to serve as an effective antibacterial agent, thus safeguarding agricultural and food safety. Xie et al. [80] showed that ZnO-NPs can effectively inhibit the growth and reproduction of *Campylobacter jejuni* (*C. jejuni*). Bratz et al. [81] showed that piglets fed a high dose of ZnO (3100 mg/kg) significantly reduced the faecal excretion of *Campylobacter coli* (*Camp. Coli*) by up to 1 log CFU/g compared to piglets fed a low (40 mg/kg) or medium (100 mg/kg) ZnO diet, which indicated that ZnO could alleviate diarrhea in piglets by inhibiting the reproduction of harmful microorganisms and promoting intestinal health.

**Figure 1:**
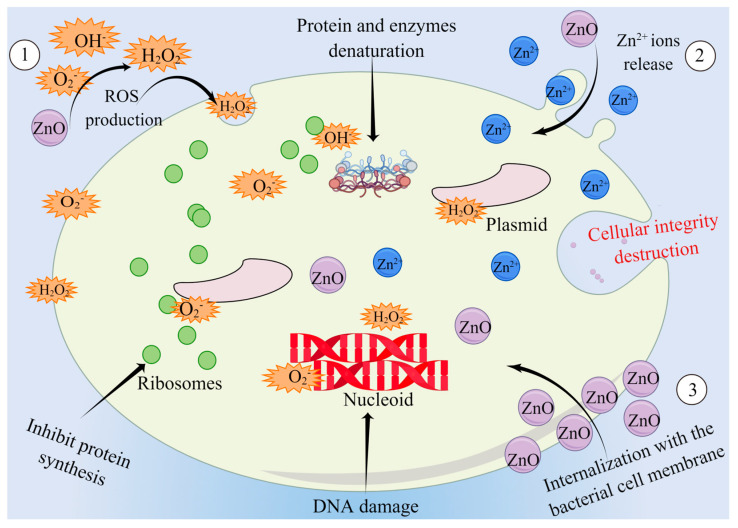
Possible mechanism of action of zinc oxide (ZnO) against bacteria (By Figdraw https://www.figdraw.com/static/index.html#/, 12 July 2023). (1) The generation of ROS, which can destroy the cellular components of pathogens such as DNA, proteins, and lipids; (2) ZnO releases Zn^2+^ ions, which can affect the enzyme activity, nucleic acid synthesis, and protein synthesis of bacteria; and (3) internalization within the bacteria cell via electrostatic forces and destroy the integrity of bacterial cells. H_2_O_2_: hydrogen peroxide; ROS: reactive oxygen species.

ZnO has been shown to have antibacterial properties against both Gram-negative and Gram-positive bacteria [82,83,84]. At present, multiple theories regarding the antibacterial mechanism of ZnO have been proposed, including the release of Zn^2+^ ions, the production of reactive oxygen species (ROS), and the direct interaction with bacterial cell membranes (Figure 1) [82,83,85]. Among these mechanisms, ROS production by ZnO is one of the most commonly reported antibacterial mechanisms [82,86,87,88]. ROS can cause oxidative stress and destroy the cellular components of pathogens, thus affecting the homeostasis of the pathogens and achieving bactericidal effects [89,90,91,92,93]. One piece of evidence suggesting that ZnO mediates its antibacterial effect through ROS production is that the presence of radical scavengers can inhibit its bactericidal effect [94]. In addition, Singh et al. [92] found that ZnO exposure can cause a large number of ROS production, protein oxidation, and DNA damage, thereby inhibiting the growth of *Deinococcus radiodurans*, which also confirmed that promoting ROS production is one of the ways ZnO exerts its antibacterial properties.

The release of Zn^2+^ is also considered to be one of the main mechanisms of ZnO antibacterial activity [82,95]. ZnO is partially dissolved in the solution, releasing Zn^2+^ ions with antibacterial activity, which can affect the enzyme activity, nucleic acid synthesis, and protein synthesis of bacteria, thus interfering with their normal growth and metabolism [95,96,97]. This theory was confirmed by Joe et al. [98], who demonstrated that ZnO initially attaches to the bacterial cell wall and then releases Zn^2+^ ions, which subsequently penetrate the cytoplasm and exert antibacterial activity. It suggests that the antibacterial effect of ZnO may be more strongly associated with ROS production than Zn^2+^ ion release, though both mechanisms could be significant depending on the context. This statement is confirmed by the study of Li et al. [99], who showed that the growth of *E. coli* was not inhibited at a concentration of about 1 mg/L, but the growth rate of *E. coli* decreased with an increase in ROS levels.

The direct contact of ZnO with bacterial cell membranes is the third possible mechanism for the bactericidal effect of ZnO [100,101]. For this antibacterial mechanism, ZnO directly interacts with bacteria externally via electrostatic forces and interferes with the electron transport chain attached to the cell wall, thereby hindering cellular metabolism and causing membrane disruption [101,102,103]. Alotaibi et al. [104] demonstrated that ZnO-NPs decreased the membrane integrity of Pseudomonas aeruginosa, leading to the increased permeability of the inner and outer membranes of the bacteria. This finding confirmed that ZnO exerts an antibacterial effect by directly interacting with the cell membrane of bacteria. It should be noted that the bacteriostatic mechanism of ZnO may be the result of various factors, and further research is needed to confirm and gain a deeper understanding of the specific mechanism. But one thing is certain, that is, ZnO has a good antibacterial effect.

### 3.2. ZnO and Intestinal Physical Barrier of Piglets

**Figure 2:**
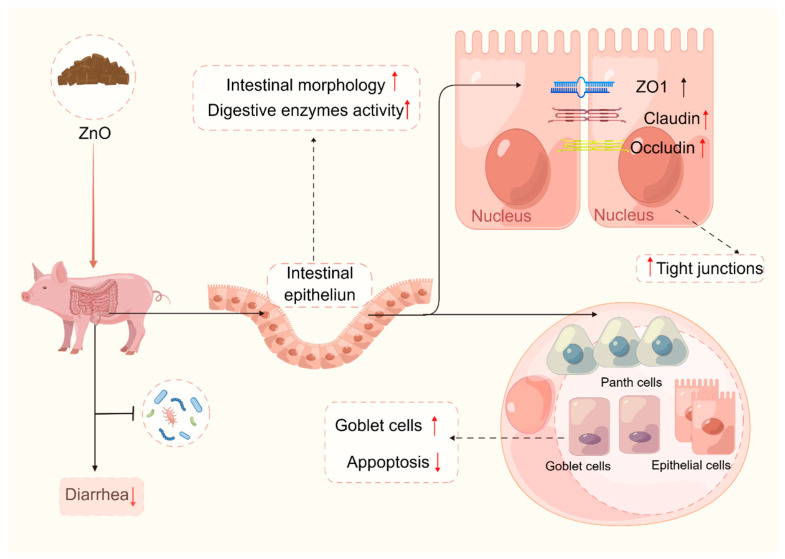
Zinc oxide (ZnO) alleviates diarrhea by strengthening the intestinal physical barrier of piglets (By Figdraw, https://www.figdraw.com/static/index.html#/, 12 July 2023). ZO-1: zonula occluden-1. “↑” stands for increase, and “↓” stands for decrease.

Maintaining the integrity of the intestinal barrier is essential for the piglets’ health as it hinders the infiltration of inflammation-causing pathogens into the tissues [105,106,107]. Weaning stress can result in damage to the intestinal mucosa, which is characterized by an increase in the permeability of the intestinal mucosa and a decrease in the expression of tight junction (TJ) proteins in the intestinal epithelial cells [3]. The impairment of intestinal mucosal integrity is the primary cause of diarrhea in weaned piglets. The addition of ZnO to the diet has short-term benefits on the intestinal physical barrier function of piglets, thereby reducing the occurrence of diarrhea in piglets [33,108,109,110]. According to previous studies, the intestinal physical barrier of piglets can be enhanced by pharmacological doses of ZnO in the following ways:

(i) Pharmacological doses of ZnO (about 3000 mg/kg) can promote intestinal development in piglets, resulting in increased villus height (VH), decreased crypt depth (CD), and increased villus height/crypt depth (VCR) [33,40,111,112,113]. Intestinal morphology is an intuitive reaction to intestinal function. Weaning may lead to intestinal mucosal atrophy and affect intestinal absorption function [114], while pharmacological doses of ZnO can restore intestinal mucosal atrophy caused by weaning stress. For example, Pei et al. [115] showed that dietary ZnO (3000 mg/kg) supplementation could improve the duodenum and jejunum VCR of weaned piglets; Yi et al. [110] showed that adding 1500 mg/kg of ZnO could increase the ileum VH of weaned piglets.
(ii) Pharmacological doses of ZnO can enhance the quantity of goblet cells in the small intestine of piglets [116,117,118], and also enhance the activities of intestinal digestive enzymes in piglets [119,120,121]. Goblet cells are secretory cells that secrete mucins and are important for animal gut health. It has been observed that weaning stress hinders the proliferation and differentiation of goblet cells [122,123,124]. Nevertheless, the supplementation of ZnO in the diet has been found to notably enhance the population of goblet cells in the villus and crypt of the small intestine of weaned piglets [116,117,118]. Intestinal digestive enzymes play a crucial role in the development and digestive capacity of weaned piglets [3]. However, weaning triggers a notable reduction in intestinal digestive enzyme activities, which is considered a significant contributor to the incidence of diarrhea in weaned piglets [125,126]. ZnO could enhance the digestion and absorption of nutrients in piglets by promoting the activities of intestinal digestive enzymes. It has been demonstrated that dietary supplementation with ZnO at an effective dose of 2500 mg/kg increased the chymotrypsin activity in the small intestinal contents of weaned piglets [119]. A study by Hu et al. [120] found that a 2000 mg/kg supplementation increased intestinal protease, lipase, and amylase activities in weaned piglets. Liu et al. [121] showed that piglets fed high doses of ZnO had a higher lipase activity in the jejunum, as well as a higher lipase and trypsin activity in the ileum than piglets fed non-ZnO diets.
(iii) Pharmacological doses of ZnO can up-regulate the expression of proteins associated with the intestinal physical barrier function of the intestine, such as zonula occludens-1 (ZO-1), Claudin 1, and occludin, while simultaneously reducing intestinal permeability in piglets [38,53,110,127,128,129]. The levels of mRNA and protein expression of TJ proteins (ZO-1, occludin, and Claudin 1), and the concentrations of diamine oxidase (DAO) and D-lactic acid in the blood, serve as reliable indicators of the extent of the physical barrier function. Previous research has demonstrated that the intestinal physical barrier function of piglets may be impaired during the weaning stage, as evidenced by the disordered distribution of TJ structures and increased intestinal permeability [7,124,130]. The dietary addition of ZnO could reduce the content of DAO and D-lactic acid in the blood, up-regulate TJ protein (ZO-1 Claudin 1, occludin) expression, and reduce intestinal permeability to alleviate diarrhea in piglets to a certain degree [38,53,110,112,127,131]. Therefore, the administration of a pharmacological dosage of ZnO has the potential to enhance the integrity of the intestinal physical barrier in weaned piglets. This is achieved through improvements in intestinal morphology, increased intestinal digestive enzyme activity, and the up regulation of the expression of intestinal TJ proteins. Consequently, this intervention effectively mitigates the risk of pathogenic microorganism invasion and successfully alleviates episodes of diarrhea (Figure 2).

### 3.3. ZnO and Intestinal Immune Barrier of Piglets

**Figure 3:**
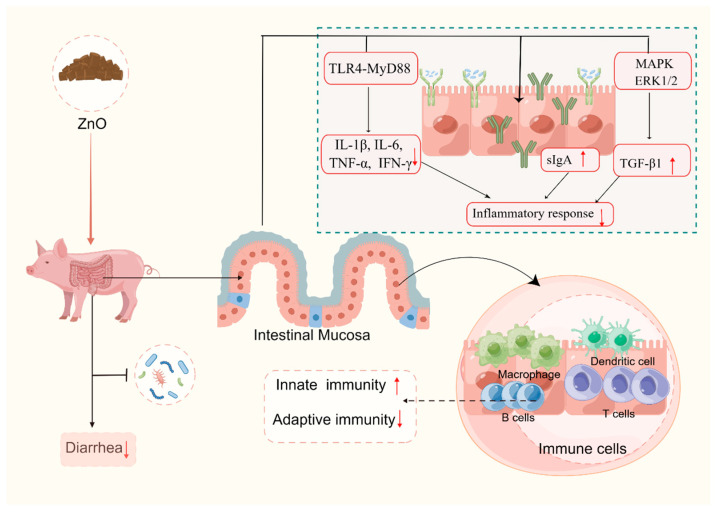
Zinc oxide (ZnO) alleviates diarrhea by strengthening the intestinal immune barrier of piglets (By Figdraw, https://www.figdraw.com/static/index.html#/, 12 July 2023). ERK1/2: extracellular regulated protein kinase 1/2; MAPK: mitogen-activated protein kinase; MyD88: myeloid differentiation factor 88; IFN-γ: interferon-γ; IL-1β: interleukin-1β; IL-6: interleukin-6; sIgA: secretory immunoglobulin A; TGF-β1: transforming growth factor-β 1; TLR4: toll-like receptor 4; TNF-α: tumor necrosis factor-α. “↑” stands for increase, and “↓” stands for decrease.

The intestinal tract serves as the primary immune organ in animals, playing a crucial role in distinguishing between exogenous antigenic stimulation and harmless antigens, thereby safeguarding against excessive sensitivity; additionally, it acts as a vital barrier, preventing the infiltration of commensal bacteria into the intestinal epithelium [132,133]. The process of weaning significantly impacts the intestinal immune function of piglets. During the post-weaning period, piglets often trigger the activation of the intestinal immune system and an inflammatory response, which can lead to intestinal barrier dysfunction [112,131,134,135,136]. Studies have shown that a high dose of ZnO supplementation can increase serum immunoglobulin (such as IgA and IgM) concentration and down-regulate pro-inflammatory cytokines (such as interleukin (IL)-1β and IL-6) in the short term [131,137,138]. The intestinal immunity of piglets may be improved by pharmacological doses of ZnO in the following ways:

(i) ZnO may promote the intestinal innate immunity and adaptive immunity of piglets. In their study, Kloubert et al. [139] conducted an analysis of the impact of ZnO on the innate and adaptive immune cells of weaned piglets. The findings revealed that the inclusion of 2500 mg/kg of ZnO in the diet resulted in enhanced innate immunity in pigs, as evidenced by heightened phagocytic activity and a slight increase in oxidative burst within the cells. Moreover, ZnO supplementation also demonstrated improvements in the adaptive immunity of piglets, characterized by an increase in T cells (CD3+) and Treg cells (CD3+CD4+Foxp3+) in the peripheral blood of porcine subjects. However, on the contrary, Oh et al. [137] conducted a study which demonstrated that the introduction of dietary supplementation containing 2500 mg/kg of ZnO resulted in a slight reduction in CD4+ T cell subsets (specifically T-bet+, RORgt+, GATA3+, FoxP3+ T cells) within the gut lymph node of piglets. Conversely, the low dose of chelated ZnO (200 mg/kg) group exhibited an increased number of T-bet+CD4+T cells (Th1) and FoxP3+CD4+ T cells (Treg), while, the low dose of nano-ZnO group (200 mg/kg) displayed elevated levels of GATA3+CD4+T cells (Th2), and RORγt+CD4+ (Th17) T cells compared to the high dose of ZnO group. These findings suggest that the immunomodulatory effects of ZnO may vary depending on the form and dosage, with lower doses of chelated or nano-ZnO potentially enhancing specific T cell subsets more effectively than higher doses of standard ZnO.
(ii) High doses of ZnO may enhance intestinal health by increasing the secretion of immunoglobulin A (sIgA), a critical component of the mucosal immune response that helps neutralize pathogens and prevent their adhesion to the intestinal epithelium [132]. For example, Han et al. [140] demonstrated an increase in sIgA concentration in the ileal mucus of the dietary group that received a dosage of 3000 mg/kg of ZnO. Similarly, Shen et al. [141] found that dietary supplementation with 2250 mg/kg of ZnO resulted in an improvement in sIgA concentration in the jejunal mucosa of piglets. These findings collectively suggest that high doses of ZnO have the potential to enhance intestinal health by stimulating the secretion of sIgA in the intestinal mucosa.
(iii) ZnO may enhance the function of the intestinal immune barrier by suppressing the expression of pro-inflammatory cytokines, such as interferon γ (IFN-γ), IL-1β, IL-6, and tumor necrosis factor (TNF-α) [33,45,142]. Weaning stress usually induces intestinal immune cells to secrete IFN-γ, IL-1β, IL-6, and TNF-α, which ultimately mediate local inflammatory response [134,135]. Using transcriptomic technology, Sargeant et al. [143] found a suppression of gene expression linked to inflammatory response in the small intestinal epithelial tissue of piglets treated with ZnO at high doses (3100 mg/kg). According to Zhu et al. [33], pharmacological doses of ZnO (3000 mg/kg) decreased the expression of genes associated with inflammation (IL-1β, and IFN-γ), and increased the expression of genes associated with anti-inflammation [transforming growth factor-β (TGF-β)] in the jejunum mucosa of piglets. Additionally, it has been demonstrated in certain studies that by inhibiting the toll-like receptor 4-myeloid differentiation factor 88 (TLR4-MyD88) signaling pathway, pharmacological doses of ZnO (2200 mg/kg) can effectively reduce the expression of inflammation-related genes, namely, TNF-α and IFN-γ [142,144]. Moreover, ZnO also induces the activation of mitogen-activated protein kinase (MAPK)/extracellular regulated protein kinase 1/2 (ERK1/2), rather than the c-Jun N-terminal kinase (JNK) and p38 signaling pathways, resulting in an increased intestinal TGF-β1 expression [145,146]. As a result, this protective mechanism helps maintain the intestinal integrity of piglets. Consequently, pharmacological doses of ZnO help maintain the intestinal health of weaned piglets by strengthening the intestinal immune barrier, which in turn helps alleviate PWD (Figure 3).

### 3.4. ZnO and Intestinal Microbial Barrier of Piglets

The intestinal tract of mammals is home to a multitude of microbial communities that form a stable symbiotic relationship with the intestinal host [3,147,148]. Clear evidence shows that dysregulation of intestinal flora can lead to intestinal dysfunction and various intestinal diseases [149,150]. Weaning is a highly stressful event for piglets as it involves a transition from easily digestible breast milk to solid feed that is more difficult to digest. This sudden change in diet leads to rapid changes in the piglets’ gastrointestinal tract, causing disturbances in their intestinal flora, which is one of the reasons for the diarrhea in piglets [31,151]. ZnO can relieve piglet diarrhea by regulating intestinal flora.

On the one hand, the antibacterial property of ZnO can make it effective against pathogenic bacteria. The commensal *E. coli* strains, mainly including enterotoxigenic *E. coli*, enteroaggregative *E. coli*, Vero- or Shiga-like toxin-producing *E. coli*, enterohaemorrhagic *E. coli*, enteropathogenic *E. coli*, diffusely adherent *E. coli*, and enteroinvasive *E. coli*, are the main pathogenic bacteria causing diarrhea in piglets [152,153]. High-dose ZnO supplementation has been shown to effectively inhibit the growth of pathogenic *E. coli* in weaned piglets [115,116,129,154,155,156,157].

ZnO, on the other hand, can promote beneficial bacteria growth. According to Christensen et al. [129], the ileal digesta of pigs was shown to contain a higher ratio of *Lactobacillus* to *E. coli* when supplemented with 3000 mg/kg of ZnO. Studies using 16S high-throughput sequencing technology have shown that the intestinal microbiota diversity and abundance of piglets could be affected by pharmacological doses of ZnO [141,158,159]. The relative abundance of lactate-producing microbes, specifically *Streptococcus* and *Lactobacillus*, in the ileal lumen significantly increased when high doses of ZnO were added to the diet of piglets, as found in studies conducted by Wei et al. [77] and Sun et al. [109]. Additionally, the relative abundance of intestinal disease-associated pathogens such as *Clostridium* [31] and *Campylobacterales* [158] decreased in weaned piglets when supplemented with ZnO in their diet. These studies indicated that the gut microbes of piglets can be regulated by high doses of ZnO.

In addition, ZnO has the potential to elevate the levels of short-chain fatty acids (SCFAs) in the intestinal contents of weaned piglets. SCFAs are primarily generated through microbial fermentation in the hindgut and provide benefits such as maintaining epithelial cell integrity, regulating immune functions, and inhibiting the growth of pathogenic microorganisms [159,160,161]. According to Zhang et al. [72], piglets fed with a diet supplemented with high doses of ZnO exhibited increased concentrations of acetate and total SCFAs in their feces. Sun et al. [109] also demonstrated that ZnO increased the acetate concentration in the colon as well as the propionate concentration in the ileum and colon. Similarly, Christensen et al. [129] demonstrated that pigs fed high-ZnO diets had higher levels of acetic and butyric acid in the ileal digesta compared to pigs fed a non-ZnO meal. Therefore, ZnO’s effectiveness in mitigating piglet diarrhea is closely linked to its ability to modulate intestinal microbiota, enhance beneficial bacteria, reduce pathogens, and increase SCFA production.

## 4. Alternatives to ZnO

In the past, the use of a pharmaceutical dose of ZnO has been widely used in pig production to prevent or reduce PWD incidence, but its use has also led to many disadvantages. Firstly, excessive or prolonged usage of a high dose of ZnO may result in the loss of ZnO benefits as well as toxic effects, such as piglet pale skin, coarse hair, and long hair, and finally, even inhibit the growth of piglets; because Zn accumulates too much in the kidney, liver, and pancreas tissues of piglets, it can lead to zinc overload [162,163,164]. Secondly, a high dose of ZnO supplementation in the diet of piglets may lead to a rise in the percentage of intestinal bacteria that are resistant to multiple drugs [165,166,167,168]. Slifierz et al. [169] showed that the use of therapeutic doses of ZnO on weaned piglets increased the resistance and prevalence of Staphylococcus aureus. Piglets supplemented with high doses of zinc oxide tended to select strains of *E. coli* that were more metal tolerant, such as those carrying the *czrC* gene, thus disrupting the potential antibacterial activity of Zn [152,170]. Thirdly, due to the low bioavailability of ZnO, most of the Zn ingested by piglets cannot be used, but enters the environment with the excretion of feces, which not only causes a waste of Zn resources but also leads to the pollution of the environment with heavy metals [36,37]. The European Union (EU) banned the use of pharmacological ZnO in feed on 26 June 2022, mainly because high doses of ZnO pose a greater risk of environmental pollution than preventing diarrhea in weaned piglets. In addition, a high dose of ZnO is also thought to cause the dysfunction of some other nutrients, such as inhibiting other trace element absorption and reducing phytase activity [171,172]. Therefore, reducing the dietary ZnO supplemental level is one of the current challenges to be addressed, and discovering alternatives to high-dose ZnO is crucial. It is suggested that controlling the flow and release of ZnO in the gastrointestinal tract could potentially improve its efficiency [9]. Additionally, some researchers believe that increasing the specific surface area of ZnO can increase the chance of ZnO contact with bacteria, thereby improving the utilization efficiency of ZnO [40,86]. Based on such assumptions or inferences, various methods have been employed to enhance the bioavailability of Zn as a substitute for high doses of ZnO, including organic Zinc [173,174], ZnO-NPs [137,175], porous ZnO [38,53], coated ZnO [109,116,176], zinc oxide–montmorillonite hybrid (ZnO-MMT) [120], and so on (Table 2).

### 4.1. Zinc Oxide Nanoparticles

The continuous and rapid development of nanotechnology has brought new development opportunities to many fields, including animal husbandry. ZnO-NPs are an example of the application of nanotechnology in animal husbandry. With a smaller size, larger surface area, and higher bioavailability, ZnO-NPs exhibit new transport and absorption properties and higher absorption efficiency [180,181,182,183]. Previous researchers have indicated that ZnO-NPs had good antibacterial properties [184,185], as well as have the ability to enhance feed utilization, improve growth performance, and alleviate diarrhea in weaned piglets [53,115,175,186]. Xia et al. [131] conducted a study where piglets were fed ZnO-NPs (600 mg Zn/kg) or traditional ZnO (2000 mg Zn/kg) for 14 days. The results revealed no significant differences in growth performance and jejunal morphology between piglets fed ZnO-NPs and those fed traditional ZnO. Moreover, the incidence of diarrhea was significantly reduced in the ZnO-NPs group and traditional ZnO group when compared with the control group, although the diarrhea incidence of piglets in the ZnO-NPs group was higher than that in the traditional ZnO group. Furthermore, ZnO-NP treatment led to the increased mRNA expression of intestinal antioxidant enzymes and TJ proteins in the jejunum, while significantly decreasing the expression levels of IFN-γ, IL-1β, TNF-α, and NF-κB in the ileum of piglets. Kim et al. [186] showed that ZnO-NPs at doses of 300 mg/kg and 500 mg/kg were equally effective as the pharmacological dose of ZnO (2500 mg/kg) in promoting growth performance, enhancing antioxidant capacity, and inhibiting the colonization of harmful intestinal bacteria in piglets. All these studies suggested that ZnO-NPs hold significant promise in substituting pharmacological doses of ZnO and promoting the overall health management of pigs in agricultural practices.

However, there are still some challenges in the application of ZnO-NPs in animal production. First, ZnO-NPs have toxic effects. In human studies, it has been confirmed that ZnO-NPs can produce ROS in cells and activate caspases3, thus, inducing cell apoptosis [187]. Second, although a large number of studies have shown that ZnO-NPs can promote growth and prevent diarrhea at lower doses than traditional ZnO, the dose of ZnO-NPs is far above the upper limit for Zn in piglet diets (150 mg/kg Zn) stipulated in the EU. As a result, it has not been approved by the European Food Safety (EFSA) for use in animal feed additives [188].

### 4.2. Organic Zinc

Organic forms of Zn, such as protein-chelated Zn [189], Zn glycinate [173,174], and chitosan-Zn chelate (CS-Zn) [140,144], can serve as viable substitutes for high-dose ZnO due to their higher bioavailability compared to ZnO. Nevertheless, the impact of organic Zn on piglet performance and diarrhea is not consistent. For example, a study conducted by Buff et al. [190] found that pigs fed 2000 mg/kg of Zn as ZnO had a higher average daily gain (ADG) and gain:feed ratio (G:F) than pigs fed the control diet or the diet containing 150 mg/kg of Zn as Zn-polysaccharide (Zn-PS), but there was no significant difference compared with piglets fed 300 mg/kg or 450mg/kg of Zn-PS in phase 1. There was no significant difference in growth performance (ADG, G:F, and average daily feed intake (ADFI)) among pigs that were given either 300 mg/kg or 450 mg/kg of Zn as Zn-PS compared to those fed 2000 mg/kg of ZnO in phase 2 and the overall period. However, the pigs that were given 300 mg/kg or 450 mg/kg of Zn as Zn-PS exhibited a 76% reduction in Zn excretion compared to those fed 2000 mg/kg of Zn as ZnO. In contrast to high dietary ZnO supplementation (3000 mg/kg), Wang et al. [173] demonstrated that a diet supplemented with Zn glycine chelate (100 mg/kg) could improve the growth performance and serum enzyme activity of weaned pigs and reduce the excretion of Zn in feces. These studies, however, disagree with the findings of Hollis et al. [191], who claimed that high levels of ZnO (2000 mg/kg) were more effective in promoting the piglet growth rate and feed efficiency than supplements containing 500 mg/kg of Zn from Zn amino acid complex, Zn polysaccharide complex, Zn methionine, Zn proteinic acid, and Zn amino acid chelate. Therefore, whether organic Zn can be used as an effective substitute for ZnO needs to be further confirmed, and more effective forms of organic Zn need to be screened.

### 4.3. Coated Zinc Oxide

Coated ZnO is a novel form of ZnO where the coating prevents premature dissolution in the stomach, allowing for release in the intestines. Many studies have shown that low doses of coated ZnO can achieve the same effect as high doses of ZnO in preventing diarrhea in piglets [117,141]. For instance, Shen et al. [141] reported that low dose coated ZnO (380 or 570 mg Zn/kg) dietary supplementation can significantly reduce the diarrhea rate in piglets by promoting intestinal development and intestinal mucosal barrier function, and there is no significant difference in the antidiarrhea effect compared with the supplementation of 2250 mg/kg traditional ZnO. Kim et al. [117] found that a supplementation of coated ZnO (100 mg/kg) in the piglet diet could promote growth performance and improve intestinal morphology, structure, and fecal consistency of piglets infected with enterotoxigenic *E. coli*, and the effect is similar to that of ZnO at the pharmacological level (2400 mg/kg). Sun et al. [109] showed that the dietary addition of 500 mg/kg of coated ZnO improved ADG and feed efficiency, reduced diarrhea, improved ileum VH, and increased occludin expression in the ileum tissue and that this impact was better than that of a high dose of ZnO (2000 mg/kg). However, some studies have found that coated ZnO is less effective in alleviating diarrhea, promoting growth, and enhancing the intestinal health of piglets compared to high-dose ZnO [177,192]. For example, Jang et al. [193] showed that coated ZnO did not influence the growth performance, fecal consistency, and digestive enzyme activity of weaned piglets. Byun et al. [177] showed that except for diarrhea rates, the ADG and feed-to-gain ratio of pigs fed 200 mg/kg of coated ZnO were lower than in those pigs fed 2500 mg/kg of ZnO. There are some problems in the application of coated ZnO in piglet production. First, the gastrointestinal tract of the piglet is immature, and the pancreatic lipase activity in the intestines is low, which may affect the release of ZnO molecules in the gastrointestinal tract. Second, the weaned piglets themselves have insufficient gastric acid secretion, and the influence of gastric acid neutralization on the effect of ZnO may not be as great as imagined, so the effect of the zinc oxide coating is not so great for piglets. Therefore, the effect and mechanism of replacing high-dose ZnO with coated ZnO needs to be further studied.

### 4.4. Porous Zinc Oxide

As a new type of ZnO, porous ZnO has a better antibacterial effect than ordinary ZnO because of its unique physical properties, such as high purity, high specific surface area, specific particle size, and excellent fluidity [194]. Although the history of the application of porous ZnO in animal production is relatively short, studies to date have shown that it can be effective in reducing piglet diarrhea at low doses [38,40,178,195]. The findings of the in vitro culture of bacteria in the stomach and jejunum of piglets demonstrated that the growth of bacteria in the stomach samples in porous ZnO medium was delayed for a longer time, and the bacterial growth decrease in media supplemented with porous ZnO was more pronounced and occurred more quickly [196]. In comparison to a traditional pharmacological dose of ZnO (3000 mg/kg) in a normal phase, Morales et al. [197] showed that a low dose of porous ZnO (150 mg/kg) considerably enhanced growth performance and health status in piglets. According to Peng et al. [38], a low dosage of porous ZnO (750 mg/kg) was found to be effective in reducing diarrhea and improving the growth of weaned piglets, and the effects of the low dosage of porous ZnO were comparable to those of high-dose ZnO (3000 mg/kg). However, it should be noted that the application of porous ZnO in livestock production is still relatively limited and requires further investigation to fully understand its application, effectiveness, and mechanism of action.

### 4.5. Other Forms of Zinc Oxide

Other novel forms of ZnO, such as microencapsulated ZnO [113,198], ZnO-MMT [120], zinc oxide/zeolite (SR-ZnO) [31], palygorskite clay adsorbed nano-ZnO (PNZ) [118], and diosmectite-ZnO composite (DS-ZnO) [112] have also been reported to have the potential to replace high doses of ZnO. For example, Grilli et al. [113] showed that the gain-to-feed intake (G/F) of piglets receiving low doses of microencapsulated ZnO (300 mg/kg or 800 mg/kg) was comparable to that of piglets receiving a pharmacological level of ZnO (3000 mg/kg) in the overall post-weaning phase. In addition, during the first 2 weeks after weaning, piglets fed with microencapsulated ZnO had similar effects on inflammatory status and ileal structure and integrity as pharmacological ZnO. Cho et al. [198] showed that low doses of microencapsulated ZnO (0.03% MZO) significantly increased final body weight, G:F, ADG, and the apparent total tract digestibility of piglets compared with high-dose ZnO (0.3% ZnO). Hu et al. [120] showed that supplementation with 500 or 750 mg/kg of ZNO-MMT is equivalent to adding 2000 mg/kg of ZnO in improving growth performance, alleviating post-weaning diarrhea, and improving intestinal mucosal integrity and digestive enzyme activity. Wang et al. [31] showed that the replacement of high-dose ZnO (1500 mg/kg) with SR-ZnO (500 mg/kg) could improve the Zn bioavailability and reduce the diarrhea rate of weaned piglets. Yu et al. [118] indicated that PNZ is not a perfect substitute, but an effective alternative to high doses of ZnO (3000 mg/kg) and antibiotics, as piglets given low doses of PNZ (700 mg/kg) show better intestinal health. Hu et al. [112] showed that the dietary addition of DS-ZnO at 500 mg/kg has a positive effect on alleviating diarrhea and promoting the intestinal barrier function of piglets, and the effect is comparable to that of high-dose ZnO (2250 mg Zn/kg).

## 5. Conclusions

**Figure 4:**
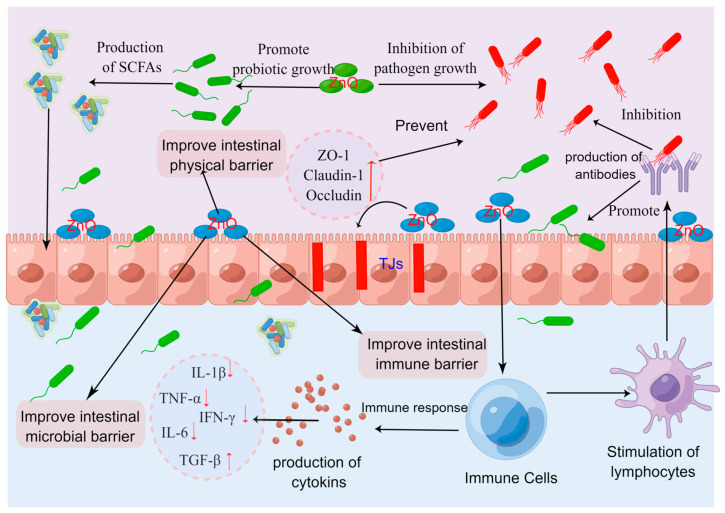
Possible mode of action of zinc oxide (ZnO) to fight against post-weaning diarrhea in piglets (By Figdraw, https://www.figdraw.com/static/index.html#/, 12 July 2023). High dose of ZnO may act through following ways: (i) direct inhibition of intestinal pathogens and modulation of intestinal microflora of piglets; (ii) improve the intestinal physical barrier function; and (iii) improve the intestinal immune barrier function. IFN-γ: interferon-γ; IL-1β: interleukin-1β; IL-6: interleukin-6; SCFA: short-chain fatty acid; TGF-β: transforming growth factor-β; TJs: tight junctions; TNF-α: tumor necrosis factor-α; ZO-1: zonula occluden-1. “↑” stands for increase, and “↓” stands for decrease.

In the past, the use of high doses of ZnO have been extensively employed in pig production to prevent and decrease the occurrence of PWD. At present, major pig-farming countries and regions such as the EU and China have clear regulations on the addition of ZnO to pig feed, especially with clear restrictions on the use of high doses of ZnO. This review presents a summary of the potential mechanisms of ZnO to prevent piglet diarrhea (Figure 4), including the following: (i) ZnO has strong antibacterial properties, which can inhibit intestinal pathogen growth, such as Enterotoxic *E. coli*, and has a positive regulatory effect on the intestinal microflora of piglets; (ii) the ability of high-dose ZnO to enhance the physical barrier of piglets by promoting intestinal morphological development, increasing digestive enzyme secretion, and enhancing the differentiation of goblet cells, while also improving the stability of the intestinal tight junction structures; and (iii) high-dose ZnO enhances the intestinal immune barrier function by improving the intestinal initial immunity and adaptive immunity, resulting in increased sIgA and anti-inflammatory cytokine secretion, as well as the inhibition of pro-inflammatory cytokines. Overall, the use of high doses of ZnO helps to alleviate PWD by promoting the overall intestinal health of piglets. Nevertheless, excessive or long-term use of high-dose ZnO has brought about many negative effects, which provides an opportunity to explore various substitutes to high doses of ZnO. So far, several innovative forms of ZnO, including ZnO-NPs, organic zinc, coated ZnO, porous ZnO, ZnO-MMT, SR-ZnO, and others, have demonstrated their potential to substitute for pharmacological doses of ZnO.

## Figures and Tables

**Table 1:** Brief description of the important role of zinc in animal health.

Species	Forms of Zinc	Doses	Major Health Benefits	Reference
Rat	ZnO-NPs	4 mg/kg BW	reproductive toxicity	Lokman et al. [54]
Mice	ZnO-NPs	20 μg/kg	IL-6↓, IL-8↓, TNF-α↓, inflammatory cells penetration↓	Chen et al. [55]
Mice	ZnO-NPs	20 μg/kg	IL-6↓, IL-8↓, TNF-α↓, inflammatory cells penetration↓	Yu et al. [56]
Rabbit	ZnO-NPs	20–80 mg/kg diet	growth performance↑, meat physicochemical properties↑, blood biochemistry parameters↑	Abdel-Wareth et al. [57]
Rabbit	ZnO-NPs	50 mg/kg diet	Digestibility of crude protein and crude fat↑, cecal *Lactobacilli* spp. ↑, serum testosterone levels↑, serum ALT↓, serum AST ↓	Abdel-Wareth et al. [58]
Rabbit	Zn-CNPs	50, 75, and 100 ppm	immune functions↑	Hassan et al. [59]
Goat	NZn	25, 50 mg/kg Zn	immune functions↑, serum hormone profiles↑	Swain et al. [60]
Calves	ZnO-NPs	50 mg Zn/kg dry matter	incidence of diarrhea↓, pneumonia↓, feed intake↑, digestibility↑, blood Zn concentration↑	Abdollahi et al. [61]
Duck	Zn-Met	0, 30, 60, and 120 mg Zn/kg diet	growth performance↑, immune function↑, intestinal health↑	Chang et al. [62]
Broiler Chickens	ZnO-NPs	20, 40, and 60 mg/kg diet	growth performance↑, nutrient digestibility↑, carcass criteria↑, liver and kidney functions↑	Abdel-Wareth et al. [63]
Broiler Chickens	ZnO-NPs	40 or 60 mg/kg diet	productive performance↑, physiological status↑	Hatab et al. [64]
Broiler Chickens	Zn-POS	0, 200, 400, 600, and 800 mg/kg diet	productive performance↑, Zn enrichment in the metabolic organs↑	Wang et al. [65]
Broiler Chickens	Gly-Zn	60 mg Zn/kg diet	growth performance↑, serum indexes↑, intestinal morphology↑	Zhu et al. [66]
Broiler Chickens	ZnO-NPs	90 mg/kg diet	body weight↑, antibacterial activity↑	Radi et al. [67]
Laying Hens	Zn-Met	40 and 80 mg Zn/kg diet	Zn contents in liver, duodenum, and jejunum↑, intestinal morphology↑, metallothionein mRNA expression↑	Li et al. [68]
Laying Hens	ZnCP	40.25 and 80.50 mg Zn/kg diet	blood iron (Fe) content↑, jejunal MT-4 mRNA abundance↑, liver Zn content↑, pancreas Zn content↑	Li et al. [69]
Laying Hens	ZnO	25 or 75 mg diet	feed intake↑, antioxidant ability↑, serum zinc status↑	Abd El-Hack et al. [70]
Weaned Piglets	ZnO	3000 mg/kg	diarrhea rate↓, growth performance↑, intestinal barrier function↑, immune function↑	Zhu et al. [33]
Weaned Piglets	ZnSO4, Gly-Zn, zinc lactate	100 mg/kg diet	growth performance↑, intestinal barrier function↑	Diao et al. [71]
Weaned Piglets	TBZC	1000 mg Zn/kg diet	zinc excretion↓, growth performance↑	Zhang et al. [72]
Weaned Piglets	HME-Zn	80 mg/kg diet	growth performance↑, antioxidant capacity↑, pancreatic enzyme activity↑, intestinal morphology↑, nutrient digestibility↑	Lee et al. [73]
Weaned Piglets	HME-ZnO	500, 1000, or 2500 ppm	digestibility of protein↑, intestinal Coliform and Clostridium↑, intestinal morphology↑	Oh et al. [74]

Gly-Zn: zinc glycinate; HME-Zn: hot-melt extrusion processed zinc sulfate; HME-ZnO: hot-melt extruded ZnO nano-particles; IL-6: interleukin-6; IL-8: interleukin-8; MT-4: metallothionein-4; NZn: nano zinc; TBZC: tetrabasic zinc chloride; Zn-CNPs: zinc-chitosan nanoparticles; ZnCP: zinc-bearing zeolite clinoptilolite; Zn-Met: zinc methionine; ZnO: zinc oxide; ZnO-NPs: zinc oxide nanoparticles; Zn-POS: zinc pectin oligosaccharides chelate; ZnSO4: zinc sulfate. “↑” stands for increase, and “↓” stands for decrease.

**Table 2:** Summary of novel ZnO as ZnO alternatives.

Novel Zinc Oxide Forms	Dose	Does of ZnO	Substitution Effect	Reference
ZnO-NPs	400, 800 mg/kg	3000 mg/kg	ADG↑, diarrhea rate↓, intestinal morphology↑, occludin↑, ZO-1↑	Wang et al. [53]
ZnO-NPs	200 mg/kg	2500 mg/kg	diarrhea rate↓, dry matter and gross energy digestibility↑, IL-6↓, IL-8↓, Succinivibrio↑	Oh et al. [137]
ZnO-NPs	300, 400, 500, 600 mg/kg	2000 mg/kg	ADG↑, diarrhea rate↓, serum ALP, IgG, SOD↑, lactic acid bacteria and total anaerobic bacteria↑, *E. coli*↓	Sun et al. [175]
ZnO-NPs	150, 300, or 450 mg/kg	3000 mg/kg	ADG↑, ADFI↑, intestinal morphology↑, diarrhea rate↓, *Escherichia colii*↓	Pei et al. [115]
ZnO-NPs	600 mg Zn/kg	2000 mg/kg	diarrhea rate↓, antioxidant enzymes↑, tight junction proteins↑, IL-1β↓, IFN-γ↓, NF-κB↓, TNF-α↓, *Streptococcus*↑, *Lactobacillus*↑, *Lactobacillus*↓, *Oscillospira*↓, *Prevotella*↓	Xia et al. [131]
zinc glycine chelate	50, and 100 mg/kg	3000 mg/kg	ADG↑, ALP↑, Cu/Zn SOD↑	Wang et al. [173]
ZnGly	400, 800, and 1200 mg/kg	2500 mg/kg	fecal score↓, TNF-α↓, Actinobacteria↑, ZIP4↑, ZnT5↑, Enterobacteriaceae↓	Jang et al. [174]
CS-Zn	50, 100 mg/kg	3000 mg/kg	serum DAO activities↓, D-lactate levels↓, endotoxin contents↓, intestinal morphology↑, sIgA↑, apoptotic cells↓	Han et al. [140]
CS-Zn	100 mg/kg	1600 mg/kg	intestinal morphology↑, *Lactobacillus*↑, *Streptococcus*, *Escherichia shigella*↓, *Actinobacillus*↓, *Clostridium* sensu stricto 6↓, propionate↑, butyrate↑, lactate↑, IL-1β↓, TNF-α↓, MPO↓, INF-γ↓	Hou et al. [144]
coated ZnO	250, 380, 570, 760 and 1140 mg/kg	2250 mg/kg	diarrhea rate↓, intestinal morphology↑, IGF1↑, ZO-1↑, occludin↑, IL-10↑, sIgA↑, microbiota richness↑	Shen et al. [141]
coated ZnO	100 mg/kg	2500 mg/kg	ADG↑, goblet cell density ↑, intestinal morphology↑, fecal consistency score↓	Kwon et al. [116]
coated ZnO	562.5 mg/kg	2250 mg/kg	diarrhea rate↓, plasma D-lactate level↓, intestinal morphology↑, occludin↑, ZO-1↑, T-AOC↑, MDA↓	Dong et al. [176]
coated ZnO	100, 200 mg/kg	2500 mg/kg	ADG↓, gain:feed ratio↓, diarrhea rate↓	Byun et al. [177]
coated ZnO	500 mg Zn/kg	2000 mg/kg	growth performance↑, diarrhea rate↓, barrier function↑, SCFAs↑	Sun et al. [109]
coated ZnO	100 mg/kg	2400 mg/kg	ADG↑, intestinal morphology↑, goblet cell density↑, fecal consistency score↓,	Kim et al. [117]
porous ZnO	200 mg/kg, 500 mg/kg	2000 mg/kg	ADG↑, ADFI↑, diarrhea rate↓, *Lactobacillus* spp.↑, *Escherichia colii*, *Clostridium coccoides*, and *Clostridium*↓, intestinal morphology↑	Peng et al. [40]
porous ZnO	500, 1000 mg/kg	3000 mg/kg	FCR↑, diarrhea rate↓	Ouyang et al. [178]
porous ZnO	500 mg /kg	3000 mg/kg	ADG↑, ADFI↑, intestinal morphology↑, diarrhea rate↓	Long et al. [179]
porous ZnO	750 mg/kg, 1500 mg/kg	3000 mg/kg	ADG↑, FCR↑, diarrhea rate↓, serum ALP↑, IgG↑, IGF-1↑, TGF-β↓, zonula occludens-1↑, occludin↑, IL-8↓, AQP3↓	Peng et al. [38]
DS-ZnO	500 mg Zn/kg	2250 mg/kg	ADG↑, ADFI↑, post-weaning scour scores↓, intestinal morphology↑, occludin↑, claudin-1↑, ZO-1↑, TNF-α↓, IL-6↓, IFN-γ↓	Hu et al. [112]
mZnO	150 or 400 mg/kg	3000 mg/kg	ADG↑, intestinal morphology↑, TNF-α↓, IFN-γ↓, occludin↑, ZO-1↑	Grilli et al. [113]
SR-ZnO	500 mg/kg	1500 mg/kg	diarrhea rate↓, serum ALP↑, zinc bioavailability↑, Campylobacters↓, Clostridium↑	Wang et al. [31]
ZnO-MMT	250, 500, and 750 mg/kg	2000 mg/kg	ADG↑, ADFI↑, fecal scores↓, intestinal permeability↓, digestive enzyme activities↑	Hu et al. [120]
alternative ZnO	300 mg/kg	3000 mg/kg	diarrhea rate↓, digestive enzyme activities↑, zinc transporter proteins↑, faecal zinc emissions↓, TJ proteins↑, mucins↑, antimicrobial peptides↑, Lactobacillus↑	Su et al. [32]
PNZ	700, 1000, or 1300 mg/kg	3000 mg/kg	intestinal morphology↑, goblet cells↑, TNF-α↓, IL-1β↓, sIgA↑, IL-4↑, MCU2↑, ZO-1↑, TLR4↓, MyD88↓	Yu et al. [118]

ADFI: average daily feed intake; ADG: average daily gain; ALP: alkaline phosphatase; AQP3: membrane channels that transport water; Cu/Zn SOD: Cu/Zn superoxide dismutase; CS-Zn: chelate of Zn^2+^ with chitosan; DAO: diamine oxidase; DS-ZnO: diosmectite-ZnO composite; *E. coli*: *Escherichia coli*; FCR: feed conversion rate; IFN-γ: interferon-γ; IGF-1: insulin-like growth factors-1; IgG: immunoglobulin G; IL-1β: interleukin-1β; IL-6: interleukin-6; IL-8: interleukin-8; MDA: malondialdehyde; MPO: myeloperoxidase; MyD88: myeloid differentiation factor 88; mZnO: microencapsulated ZnO; NF-κB: nuclear factor kappa-B; PNZ: palygorskite clay adsorbed nano-ZnO; SCFAs: short-chain fatty acids; sIgA: secretory immunoglobulin A; SOD: superoxide dismutase; SR-ZnO: zinc oxide/zeolite; T-AOC: total antioxidant capacity; TGF-β: transforming growth factor-β; TJ: tight junction; TLR4: toll-like receptor 4; TNF-α: tumor necrosis factor-α; ZIP4: IRT-like protein 4; ZnGly: zinc glycinate; ZnO-MMT: zinc oxide–montmorillonite hybrid; ZnO-NPs: zinc oxide nanoparticles; ZnT5: zinc transporter 5; ZO-1: zonula occluden-1. “↑” stands for increase, and “↓” stands for decrease.
